# The Remote and Proximate Cause of Bilious Fevers of the South and West, Considered in the Light of Liebig’s Theory of Animal Heat

**Published:** 1846-08

**Authors:** Samuel G. Armor

**Affiliations:** Rockford, Ill.


ARTICLE II.

*The remote and proximate cause of Bilious Fevers of the South and West, considered in the light of Liebig's Theory of Animal Heat.* By SAMUEL G. ARMOR, M. D., of Rockford, Ill.

The science of Medicine should be strictly eclectic. Its object should be to glean well established facts from every department of Nature. It should be the great aim of the intelligent physician, therefore, to divest the sciences of their isolated character. By studying them in this manner, we lose their real end and object, and are apt to forget the great truth of the unity of all science. If the question were propounded as to the true object of science, the answer would be very indefinite, and, doubtless, by no means uniform or consistent; each would assign the special end of the one he cultivates, without making a single deduction from the progress of his speciality, to that of other specialities. Having studied the elements of the sciences in themselves, we should study them in their relations, and this must end in establishing the most intimate ties between the most distant sections of the vast net work of the sciences.

The science of Medicine, practically considered, is perhaps more directly and largely indebted to modern improvements in Chemical science, than all others combined. Chemical researches into the composition of the fluids and solids of the animal body, and comparative examinations of them under the differences of age, sex, climate, mode of life, and the various modifications of disease, have thrown great light both on the healthy and disordered actions of our frame; and by discovering what particular ingredients existed in undue proportion, they have also suggested the means of relief to be often the internal administration of suitable chemical remedies. The views, for instance, respecting the nature and treatment of calculus diseases, are truly chemical; and for the theory of diabetes we are also indebted to Chemistry. In the department of Toxicology we are also largely indebted to the progress of this science. The specification and therapeutical application of remedies, the knowledge of the action of the different kinds of poisons, and of their antidotes, are learned from chemical research and experiments. The object of this paper is to present some thoughts founded on the acknowledged principles of Chemical science, in relation to the operation of that intangible, undefined and undefinable something, called "Miasma," together with the rationale of the therapeutic action of Quinine as its antidote. The suggestions I have to make are founded principally on the research and observation of that distinguished and able chemist, Prof. Liebig, and so far as relates to his theory of Animal Heat, I claim no originality. His elaborate and ingenious argument on this long mooted question may be briefly summed up in this proposition: "The combination of a combustible substance with oxygen is, under all circumstances, the only source of animal heat, and the combination of oxygen with carbon is always accompanied by the disengagement of heat." This being assented to, it follows that the amount of oxygen consumed bears a relation, from necessity, to the amount of heat liberated, and this depends upon the temperature of the surrounding medium. Nature requires that the quantity of inspired oxygen shall increase or diminish in proportion to the temperature of the external air. The capability of this physiological adaption to all climates, appears to be peculiar to Man, and it is a wise provision of nature, that he has been endowed with this power. It enables him to pass into all the climates of the earth; to reside beneath the pole or the equator; to live under a burning sky or on an ice-bound soil; and to inhabit regions where the most hardy animals can scarcely exist. But this change of climate and season are necessarily connected with corresponding changes in the action of vital force, and this depends upon the laws which govern the production of animal heat. The blood of the inhabitants north of the Arctic Circle has a temperature as high as the inhabitants of the Torid Zone; in the one case the temperature is nearly equal to that of the blood, in the other from 75° to 90° lower. The loss of heat from external temperature, in the one case, must be supplied by an increased quantity of inspired oxygen, and this increased proportion of oxygen, found in a dense and cold atmosphere, must have a combustible substance with which to unite. It follows, then, that the quantity of oxygen must be increased in the same ratio with combustible matters. The appetites, practices, and customs of tribes and nations of different climates, throw much light upon this subject. If we should take exercise in a cold atmosphere in winter, or go naked like savages, we would require food containing carbon and hydrogen in the same increased ratio, and could eat with impunity, (as is the case with northern tribes,) large quantities of flesh, tallow, oil, &c. But let us take another and a physiological view of this question. An Englishman accustomed to good dinners goes South—finds his appetite fails him—uses stimulating condiments to excite it. But the temperature of the atmosphere does not require the consumption of a large quantity of oxygen, and nature, true to her established laws, has not furnished a large amount. The result is, that a large portion of the carbon thus introduced into the system, is unconsumed. Hence the pathological effects. Compounds, rich in carbon, appear in the urine, which, in consequence, acquries a brown color. It is also detected in the peculiar odor of the perspiration, which, says Prof. Liebig, contains much carbon. The dark and tawny color of the skin is also referable to the operation of this law of Animal Chemistry. Have we not, then, many facts drawn from the laws of Chemical relation which throw much light on diseased action? And how beautifully those laws harmonize with the general economy of Nature? If the same quantity of oxygen was consumed in the warm summer months, when the surrounding medium did not rapidly liberate the heat thus generated, the result would be excessive febrile excitement, and the destruction of our organism by the action of oxygen. The practical inference from this is, that in warm seasons of the year, or in a southern climate, we should use sparingly of carbonized articles of diet. To use large quantities of flesh, oil, and fatty substances, when the surrounding medium does not require the production of so much animal heat, and when in fact, nature has not provided, in the same proportion, a counteracting or consuming agent, would be, not to create a proportional amount of animal heat, because that is governed by the amount of oxygen consumed, but to increase in undue proportion, one of the chemical ingredients of our physical structure.

What are the pathological effects of this excess of non-azotized matter? And how does it operate in the production of fever? These are interesting questions to the Southern and Western practitioner, and deserve our closest investigation.

Prof. Liebig thinks he has settled the proposition in his "Animal Chemistry," that the Liver has a remarkable affinity for carbonaceous substances. He states that in inflammation of the liver, we find the blood loaded with fat and oil. This observation is corroborated by Dr. Stevens, to whom we are greatly indebted for much careful analytic investigation. Assuming this to be a fact, does this condition of the circulating mass give rise to the inflammatory action of the liver? or does this deterioration of the blood, and consequent disturbance of functional harmony, arise from perverted glandular action, growing out of a primary lesion of nervous energy? Here the long mooted question of fluidism and solidism—difficult to settle because from their direct and intimate connection—we cannot conceive of one being affected without implicating the other,—again presents itself. While we shall indulge in a little interrogative speculation, as to primary impression, we shall not attempt to separate this direct physiological connection.

Again, Liebig asserts it, as a universal fact, that the nitrogenized constituents of vegetable food have a composition identical with that of the constituents of the blood. He also asserts it as a well established truth, that when animals are fattened on food destitute of nitrogen, certain parts of their structure only increase in size. He instituted experiments on animals, and noted the result. Examples of geese are given, fattened in the method above alluded to. The result was, enlargement of the liver to three or four times its natural size, and a structure soft and spongy. From these facts we present the apriori argument, that an excess of carbon lies at the foundation of Hepatic diseases.

But the question may be asked here, why is it that Bilious and Intermittent fevers prevail more in some localities than others, in the same latitude and in the same season of the year? Our answer to this interrogatory is, 1st. In the warm summer months a predisposition to those diseases exists in any location. 2d. Every supposed favorable condition for the generation of Miasma is also favorable for the generation of carbonic acid gas. Chemical analysis establishes the fact that carbon enters very largely into the constituents of all black vegitable loam. This character of soil is generally found in rich river bottoms, or in low marshy countries where those types of fever prevail. The decomposition of vegetable matter also furnishes an additional source of this element. We have then, 1st. The predisposition. 2d. The soil and decay of vegetable matter impregnating the atmosphere, which we are constantly inhaling, with a double proportion of the very same agent which develops the predisposition. Add to these exciting causes, such as sudden changes of the temperature, operating on an already relaxed and debilitated condition of the animal frame, want of healthy elimination and depuration, and a consequent interruption of the harmony of function, so essential to a normal condition of the fluids and solids of the body, and you have our answer to the interrogatory.

Another interesting question presents itself in connection with this subject. How do the preparations of Cinchona, operate in arresting a paroxysm of fever? Do they operate primarily on the blood by changing its chemical relations, which have been disturbed by the introduction of a foreign body? or on the Nervous Centres, and through them on the fluids of the body? The satisfactory solution of this question, it will be at once perceived, depends much on the settlement of that other question as to *primary* impression. If we subscribe to the clearly elaborated views of Dr. Stevens, and other able pathologists of the present day, that "in those fevers which arise from marsh miasmata, or from contagion, a diseased condition of the blood is the first which occurs in the train of symptoms, and the immediate cause of those which follow," then it is rational to suppose that our curative agents operate directly on this proximate cause. This view appears to be sustained also by the fact, that quinine is a very highly nitrogenized vegetable principle, having a composition identical with the constituents of healthy blood, and thereby furnishing the direct antidote to this perverted circulating mass. We might sustain this theory by much plausible argument. We might reasonably contend that the temperature, the vital activity, the renovation of all the organs are constantly maintained at the expense of arterial blood; that a normal condition of the solids, depends on the natural response of every surface, of every organ, to its natural, its healthy, and its appropriate stimuli. If the blood, therefore, be the fountain of life from which all the organs and tissues of the body are immediately formed, it follows that whatever deranges the nutritive process, by which the healthy condition of vital action is maintained, will produce derangement of function, abnormal action and disease. In its healthy state, blood contains definite quantities of oxygen, hydrogen, azote and carbon. An increased or diminished proportion of any of these constituents gives rise to morbid states of the vital current. Its vitality being diminished, every organ and every fibre suffers. The brain and nervous system, no longer supplied with good arterial blood, fail to perform their healthy functions. Hence the loss of balance between healthy nervous and vascular excitement. The harmony and order of the Physiological state, is rapidly succeeded by the "horrible discord" of the Pathological. A shattered and depressed condition of the nervous system exists; respiration is diminished; temperature of the body is reduced; there is loss of sensation, impaired memory, head ache, loss of appetite, nausea &c., followed by reaction, with all the characteristics of fever.

If carbonized substances, therefore, such as fat, oil, tallow, caffeine, asparagine, &c., as well as all non-azotized articles of food, are, as expressed by Prof. Liebig, "food for the liver," furnishing the elements by the presence of which that organ is enabled to perform its functions, may we not with equal justness conclude, that the brain and nervous matter are formed in a manner similar to that in which bile is produced, but requiring a different element. If the propositions be true, as set forth by this learned author, is it not a reasonable conclusion, that a change is effected in the composition and quality of the constituents of the blood, by the introduction of a highly nitrogenized compound, and that quinine operates as a direct stimulent or sustenant to the brain and nervous system, by entering the circulation, changing its chemical relations, and becoming converted into constituents of brain and nervous matter, and into organs of vital energy, from which the organic motions of the body derive their origin? If it be an undeniable truth, as alleged, that the substance of the brain and nerves, is drawn from the elements of nitrogenized vegetable food, and that a depressed and perverted condition of nervous energy, grows out of a vitiated circulation, is there any thing absurd in the opinion that other constituents of vegetables, containing the chemical elements of medulary and nervous matter, may be applied to the same purpose?

In reviewing some old interrogatories, and raising some new ones, do not accuse me of wishing to deal in vain and idle speculations. I am in search of *facts.* A junior in my profession, I submit these suggestions to the criticism of my seniors. If I err, attribute it to my zeal to abandon speculation and hypothesis, for facts adduced from experiment and observation. This is the glory of Medical Science: the medical man is the man of induction, having opinions based on a broad range of facts and observation.

In presenting these views, I have no theory to sustain. To the *Chemical School* of Physiology we are largely indebted for many interesting facts in our Science; yet to the very first objection urged by the school of *Vitalism*, that organic and inorganic nature are distinct in many of their most essential attributes, no one can refuse his assent. We would not therefore, wrap up our Art in a kind of learned mystery, on the one hand, nor submit man, as charged by Prof. Paine, to the same laws which govern a stone, on the other. In the Investigation of Nature's laws, the man of Science should strive, with Lord Bacon, "to make wonders plain, not plain things wonders." What a noble rule for Physicians! By collecting facts they may serve to explain the train of phenomena, and devise a Theory which will develope the rationale of our Western fevers. Every fact, every well attested observation, will give us motives for searching into analogies, suggest new modes of observation and experiment, and may serve as a scaffold for the erection of general laws. Our science rests on many interesting and valuable facts; yet it must be confessed that our knowledge in the line of inquiry here presented, when compared with that of which we are ignorant, is extremely defective. An ample harvest for future laborers, yet presents itself. Let us strive to enrich our profession by gathering facts in this field of research.

